# PCR in Forensic Science: A Critical Review

**DOI:** 10.3390/genes15040438

**Published:** 2024-03-29

**Authors:** Caitlin McDonald, Duncan Taylor, Adrian Linacre

**Affiliations:** 1 College of Science & Engineering, Flinders University, GPO Box 2100, Adelaide, SA 5001, Australia; caitlin.mcdonald@flinders.edu.au (C.M.); adrian.linacre@flinders.edu.au (A.L.); 2 Forensic Science SA, GPO Box 2790, Adelaide, SA 5001, Australia

**Keywords:** denaturation, annealing, DNA amplification, polymerase chain reaction, STR amplification

## Abstract

The polymerase chain reaction (PCR) has played a fundamental role in our understanding of the world, and has applications across a broad range of disciplines. The introduction of PCR into forensic science marked the beginning of a new era of DNA profiling. This era has pushed PCR to its limits and allowed genetic data to be generated from trace DNA. Trace samples contain very small amounts of degraded DNA associated with inhibitory compounds and ions. Despite significant development in the PCR process since it was first introduced, the challenges of profiling inhibited and degraded samples remain. This review examines the evolution of the PCR from its inception in the 1980s, through to its current application in forensic science. The driving factors behind PCR evolution for DNA profiling are discussed along with a critical comparison of cycling conditions used in commercial PCR kits. Newer PCR methods that are currently used in forensic practice and beyond are examined, and possible future directions of PCR for DNA profiling are evaluated.

## 1. The Polymerase Chain Reaction & Forensic Science

### 1.1. The General Principles of PCR

The polymerase chain reaction (PCR) is a fundamental technique that amplifies specific regions of deoxyribonucleic acid (DNA) via an enzymatic reaction. Amplification using PCR requires five key components: deoxynucleotide triphosphates (dNTPs), thermostable DNA polymerase, template DNA, primers, and a buffer containing potassium and magnesium [1,2,3]. The high specificity of PCR can be largely attributed to the sequence-specific primers present in the reaction and stringent cycling conditions employed. PCR programs involve the same three basic steps of denaturation: heating to dissociate the double strands of the DNA molecule; annealing to allow primers to bind to their complementary target sequence; and extension, where the sample is heated to a temperature slightly above annealing that is optimal for DNA polymerase to synthesise the new double-stranded molecule [2,3,4,5,6]. These three steps are cycled through until sufficient target DNA is produced that can be detected.

### 1.2. Historial Significance and Applications of PCR in Forensic Science

In 1981, the entire human mitochondrial genome was sequenced and published [7]. This sequence, known as the Cambridge Reference Sequence (CRS), was quickly adopted by geneticists and became a widely used reference point for many mitochondrial DNA (mtDNA) studies [7,8]. The development of DNA fingerprinting in 1985 [9] and the rise of the PCR in forensic biology during the early 1990s [10,11,12,13] revolutionized forensic casework and significantly increased the tissue types submitted for forensic analyses. Initially, forensic DNA profiling was conducted using restriction enzymes that targeted hypervariable regions within the human nuclear and mitochondrial genomes called restriction fragment length polymorphisms (RFLPs) [14]. However, the introduction of PCR to target and amplify specific hypervariable regions of the human genome marked the beginning of the DNA profiling era. The first instance of PCR being used in a criminal trial was in 1986 for the case of *Pennsylvania v. Pestinikas*, where the amplification of the Human Leukocyte Antigen (HLA) DQα was presented in court [15].

As a result, in 1991 the PCR was successfully used to amplify two regions of the mitochondrial genome, HV1 and HV2, from skeletal remains using oligonucleotide hybridization [16]. The remains were identified to belong to a 3-year-old child that had been reported missing by their parents in 1984, thus marking the first published instance of human identification using mtDNA analysis [16,17]. In 1992, the PCR was successfully used to amplify a single variable number tandem repeat (VNTR) locus within the human nuclear genome, called D1S80 [12], and in 1993 the same was done for an amplification restriction fragment polymorphism (ARFP) locus spanning the human major histocompatibility complex (MHC), also known as the HLA, called DQα [10]. Shortly after, in 1994, nine sets of human remains discovered in a Russian forest were identified to be the Romanov family, the Russian Imperial Family who had been assassinated in 1918, through extensive mtDNA and short tandem repeat (STR) analysis [18]. Later that same year, Kimpton et al. [19] published a quadruplex PCR method, which amplified four tetrameric STRs in a single reaction and produced DNA profiles that could provide a limited power of discrimination between individuals. For a significant amount of time, using restriction digests to isolate RFLPs was favored over STR PCR assays because the discrimination power afforded by RFLP analysis was significantly higher than that afforded by the four STR loci [20]. However, the advent of STR multiplexing PCR for DNA profiling allowed the introduction of additional hypervariable STR loci such that the technique of DNA profiling quickly superseded RFLP analysis to become the cornerstone of the forensic biology discipline. Importantly, the PCR cycling conditions used in these landmark publications are still largely the same as those used for DNA profiling today.

### 1.3. Driving Factors of PCR Evolution in Forensic Science

A phase of rapid change and discovery, driven by the need for standardization, reliability and reproducibility in forensic DNA analysis, occurred between 1990 and 1995. This resulted in the formation of the European DNA Profiling Group (EDNAP), the European Network of Forensic Science Institutes (ENFSI) and the Scientific Working Group on DNA Analysis Methods (SWGDAM) (originally TWGDAM), organizations that aimed to standardize techniques and ensure equal justice outcomes from biological examinations from different forensic laboratories. In the years following the first national DNA databases were established in the United Kingdom [21], other countries in Europe [22] and the United States [23]. DNA databases are compilations of DNA profiles obtained from references of individuals involved (or suspected of being involved, depending on the jurisdiction) in criminal activity (the exact criteria for inclusion on a database varying by country and state), or obtained as unknown profiles from casework. Such compilations allow DNA profiles from unknown sources, obtained from exhibits of forensic relevance, to be searched against the profiles in the database for a potential match. These databases required a set of standard STR markers to be used by all participating laboratories to allow profiles to be compared, and quality assurance so that all profiles uploaded would be reliable between laboratories [23,24,25]. To avoid coincidental matches, additional hypervariable loci were added to the multiplex STR systems [26,27], with separation initially on polyacrylamide gels and then capillary electrophoresis for DNA profiling becoming standard practice. However, despite the inherent value of using DNA databases in criminal investigations, the ethical and legal implications associated with their use posed considerable challenges for their implementation [28,29,30]. Over the last two decades, substantial progress has been made in this arena, with many countries now having their own national DNA databases. As a result of the continued development and expansion of standard STR marker sets and laboratory standardization, international databases, such as the INTERPOL DNA database, now exist and are routinely used in forensic investigation [31].

**Figure 1:**
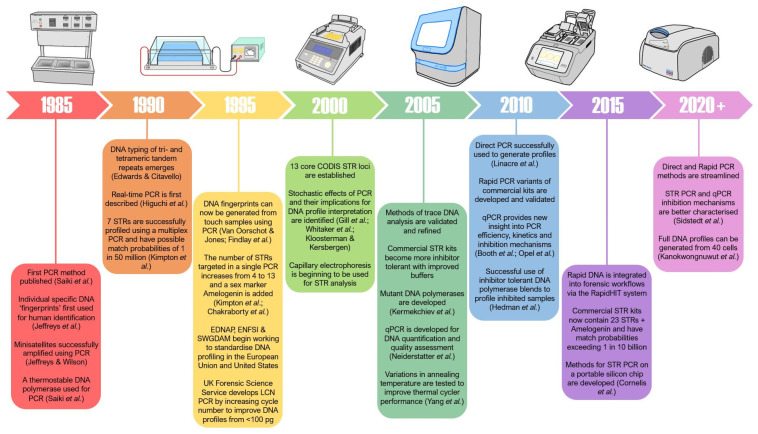
A timeline of PCR evolution within forensic science from 1985 to the present day (2023). 1985–1990 [4,5,14,49], 1990–1995 [19,50,51], 1995–2000 [52,53,54,55,56], 2000–2005 [57,58,59], 2005–2010 [60,61,62], 2010–2015 [63,64,65,66], 2015–2020 [67], and 2020+ [68,69].

The rapid developments of PCR and DNA profiling in more recent years can largely be attributed to the need for high sensitivity, specificity and reproducibility in forensic techniques. The optimal amounts of DNA recommended by manufacturers to generate probative STR profiles have substantially decreased, from 2 ng in 1995 [19] to as little as 0.4 ng in current STR kits [32,33,34,35]. However, forensic samples are commonly submitted for PCR with far lower than optimal amounts of DNA, pushing the boundaries of PCR [36]. Importantly, as the sensitivity of these techniques has increased, the number of trace DNA samples submitted to operational laboratories for analysis has also increased dramatically [37,38,39,40]. Despite the improvements in machine and PCR assay sensitivity in recent years, the success when generating DNA profiles from these trace samples remains poor [41,42,43,44,45,46,47,48]. Given the volume of trace samples that are now routinely submitted for analysis, the time and monetary costs associated with processing these samples only to obtain very little genetic information is a strain on operational laboratories around the globe. Examples of the developments in forensic genetics are shown in Figure 1.

### 1.4. Current PCR Workflows in Forensic Science

**Figure 2:**
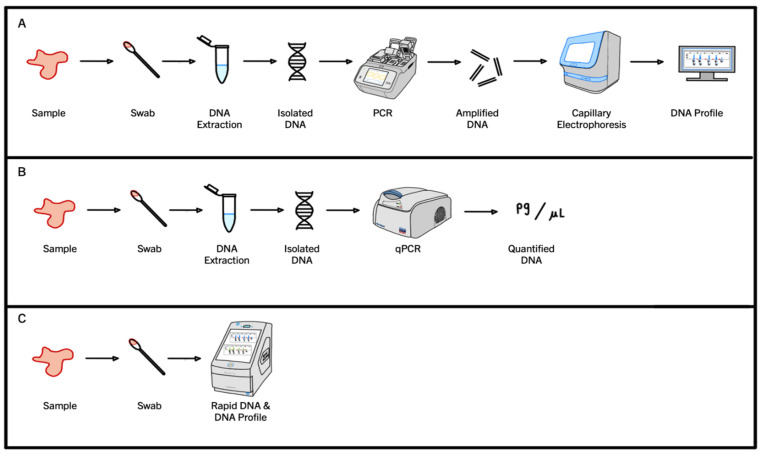
A comparison of the steps required to go from the initial crime scene sample to the final product via three different PCR methodologies: traditional PCR (**A**), quantitative PCR (qPCR) (**B**), and Rapid DNA (**C**).

The PCR process for DNA profiling, also known as STR PCR, utilizes a specialized form of chemistry where fluorophores attached to primers become incorporated into amplicons as the reaction progresses [33,54,70,71,72,73,74,75]. Once the STR PCR is complete, the amplified product is detected during capillary electrophoresis using the incorporated fluorescence tags. A visual representation of this traditional STR PCR workflow is shown below in pathway A (Figure 2).

However, in accredited forensic laboratories, the DNA present in a sample must be quantified before it progresses to STR PCR for DNA profiling. The dynamics of quantitative PCR (qPCR) are substantially different to those of an STR PCR. Commercially available qPCR kits, such as QuantiFiler Trio (Thermo Fisher Scientific, Waltham, MA, USA) or Investigator Quantiplex Pro (QIAGEN, Hilden, Germany), use TaqMan^®^ probes [75,76]. These probes are labeled with two fluorescent dyes: a reporter dye at the 5′-end and a quencher dye at the 3′end [23,77]. When the probe is intact (prior to polymerization), the two dyes are in close proximity due to the small size of the probe. The presence of the quencher dye so close to the reporter dye means the fluorescence of the reporter dye is suppressed due to an energy transfer occurring between the two. This energy transfer is based on fluorescent resonance energy transfer (FRET) principles, where the efficiency of FRET is dependent on the inverse sixth power of the intermolecular separation of the two dyes [77,78]. When a qPCR program is carried out, the probes bind to the target sequences during the annealing stage and the reporter dye fluorescence remains suppressed. However, during the extension stage, the 5′-exonuclease activity of DNA polymerase displaces the bound TaqMan probes, and the reporter dye attached to the 5′-end is separated from the quencher dye at the 3′-end [23,77,78]. The separation means the energy transfer that suppressed fluorescence when the two fluorophores were in close proximity is no longer occurring and the reporter molecule can now fluoresce [23,77,78]. The amount of fluorescence detected correlates directly with the amount of DNA present in the reaction, as the greater the amount of DNA, the more reporter dye molecules are cleaved, and the greater the fluorescence detected. Typically, as the qPCR program progresses, and the DNA increases exponentially, an amplification curve is generated using these fluorescence data, which (at the end of the qPCR program) are then used to calculate a DNA concentration. If a sample is found to contain a sufficient amount of DNA, such that useful genetic information could be obtained from it, then that sample can progress to STR PCR and a DNA profile can be generated. A visual representation of a standard qPCR workflow is shown below in pathway B (Figure 2).

The third PCR workflow that is often used in forensic science is Rapid PCR. Rapid PCR uses the same fluorescent primer chemistry as STR PCR, but has specialized instruments, PCR programs and reaction chemistries that allow DNA profiles to be generated in a much smaller time frame. Rapid PCR protocols skip the DNA quantification step but are capable of generating DNA profiles quickly using shortened PCR programs. Thus, this workflow allows informative DNA profiles to be generated quickly, but to do so samples must contain large amounts of high-quality template DNA, such as those obtained from reference swabs [73,79]. A visual representation of a standard Rapid PCR workflow is shown below in pathway C (Figure 2).

## 2. Fundamental Factors of the Polymerase Chain Reaction

### 2.1. PCR Variants—Uniplex and Multiplex PCR

While this basic formula for DNA amplification has been largely conserved since it was first conceptualized in the 1980s, some modifications have been made to the PCR process to allow it to be applied to a broad range of fields, such as microbiology, medicine and forensic science. Perhaps most notable of these developments is the advent of multiplex PCR. Initially, PCRs were only capable of amplifying one specific fragment of DNA in a single reaction [1,2,3], which was sufficient for many applications in clinical and medical diagnostics. However, in forensic science, PCR is used in an attempt to collect as much genetic information from a crime scene sample as possible. This is important because the more information that can be collected about DNA within an evidence sample, the more discrimination power there is to distinguish a donor of DNA from a non-donor, which then can be used to inform investigators about potential exclusions and contributors to a sample. To achieve this, multiplex PCR is conducted instead of uniplex PCR. Unlike uniplex PCR, multiplex PCR allows multiple regions of DNA to be amplified in a single reaction [80]. This helps reduce the amount of DNA extract needed to produce a DNA profile and the PCR reagents required to produce informative genetic data, while also increasing sample throughput when compared to uniplex PCR [81,82,83]. However, the stringency of the cycling conditions used for multiplex PCR is much higher than for those used for uniplex PCR.

The significance of multiplexing in the realm of DNA evidence is most apparent in the highly discriminatory DNA profiles obtained from the DNA amplified using multiplex PCR. The amplification of more than 20 hypervariable regions of the human genome, known as short-tandem repeats (or STRs), in a single reaction for DNA profiling imposes some restraints on the cycling conditions that can be used. As the number of regions targeted during multiplex PCR increases, so too does the number of primers needed in a reaction to ensure that the new target regions are amplified from all human populations around the world [70]. All these primers also need to be able to bind to their complementary sequence and avoid mismatching; thus, to ensure correct amplification, the annealing and extension temperatures used in multiplex PCR are highly stringent and well validated.

### 2.2. Factors Influencing PCR Cycling Conditions

There are two key factors that dictate the success of a PCR program: primer melting temperature and DNA polymerase processivity. The temperature at which the annealing step occurs is dictated by the primer melting temperature (T_m_) [62]. The T_m_ value for a pair of primers is indicative of the stability of the double-stranded DNA (dsDNA), and is determined by the temperature at which one half of the dsDNA will dissociate. Longer primers and those that are rich in guanine and cytosine have been found to have higher T_m_ values due to greater amounts of energy being required to break the bonds between the primer and the target DNA [84]. The T_m_ value for a set of primers can be determined using specialized software programs [85,86,87]; however, the methods used to calculate T_m_ values differ slightly between programs, which means the calculated values differ slightly between programs. Importantly, for multiplex PCR (which involves many primers), all primers in a reaction must have similar annealing temperatures, and thus T_m_ values, but minimal overlap with each other to minimize primer interactions [23,88]. Primer–primer interactions are detrimental to PCR because primers with regions of high complementarity will preferentially bind to one another and form primer dimers or hairpin structures, rather than bind to the template DNA, which will significantly reduce the amount of target DNA amplified [23,89]. Due to the number of independent primer annealing events that must occur simultaneously in multiplex PCRs, the temperature and timing of the annealing step is often optimized and extensively validated for each specific set of primers. To ensure all STRs are amplified in approximately equal amounts to produce balanced DNA profiles, the primers used in commercially available STR PCR kits are often manipulated to ensure effective amplification. An example of this is primers being made smaller or larger, which will decrease or increase the binding specificity and alter the T_m_ value to align with the ideal annealing temperatures for a given STR kit. Therefore, the success of DNA amplification via PCR is largely dependent on the suitability of the annealing step for the set of primers present in the reaction.

The processivity of a DNA polymerase refers to the speed at which the polymerase can synthesize a complementary DNA strand, and is measured as the number of dNTPs incorporated in a single association/dissociation event [90]. The timing and temperature of the extension step of PCR is heavily influenced by the processivity of the DNA polymerase used. The importance of polymerase processivity in PCR cycling conditions is perhaps most evident in the context of DNA profiling. While the timely amplification of DNA is ideal for all applications of PCR, the most useful and informative DNA profiles are those that contain all the target STRs (alleles) in approximately equal amounts. To promote total and equal polymerization of the target STRs, the time allowed for the extension step of PCR needs to account for the size of the target sequences and the speed at which the DNA polymerase can synthesize the amplicons. However, it is well understood that the enzymatic activity of DNA polymerase does not remain constant across a PCR run [1,91]. In the early cycles of PCR, enzymatic activity is high as the enzyme is “fresh”, there are sufficient amounts of catalyst in the reaction vessel, and there are only a few copies of template DNA to be amplified. After a few cycles, the DNA polymerase will have started to lose enzymatic activity through the heat denaturation that is occurring during the denaturation steps of the PCR [91,92]. As the PCR progresses, the available catalysts in solution decrease, the amount of template DNA continues to increase exponentially, and the repeated heating to denature the dsDNA strands continues to reduce polymerase enzymatic activity. Therefore, accounting for enzyme processivity when PCR programs are designed is essential. This involves ensuring there is sufficient time for polymerization to occur, and that complete polymerization is encouraged as enzymatic activity decreases across the PCR.

## 3. Evolution of the Polymerase Chain Reaction

### 3.1. Evolution of PCR Cycling Conditions in Forensic Science

#### 3.1.1. Short Tandem Repeat Profiling

While PCR’s cycling conditions have seen some minor variation since it was first used in forensic science in 1993, the process of amplifying STRs for DNA profiling has remained largely the same. This conservation can be largely attributed to the significant increase in STR loci targeted for DNA profiling. The introduction of more STR loci to the PCR multiplex mixtures meant the number of primers required for amplification of these highly polymorphic regions increased, and with them the stringency of the annealing stage [70]. With the addition of new STR loci came the addition of more primers to the PCR to ensure that the new loci would be amplified for all human populations around the world [70]. These primers also needed to be able to bind to their complementary sequence and avoid mismatching; thus, to ensure correct amplification, the PCR cycling conditions used today are highly stringent. As a result of this stringency and in order to further improve the quality of DNA profiles obtained from challenging samples (i.e., inhibited and trace material), changes were made to other elements of the amplification process (i.e., buffers, enzymes, instruments).

While there have been minor changes made to recommended cycling conditions between kits (Table 1), the same largely invariant conditions are often used in the validated commercial STR multiplex kits. In an effort to increase the success of DNA profiling for low-template DNA samples, the PCR cycle number can be increased by one to five cycles [57,58,59]. Importantly, increasing the cycle number in this manner has also been found to further amplify stochastic characteristics, which can make profile interpretation and deconvolution substantially more difficult [36,48,58]. Furthermore, while the addition of extra cycles of amplification has proven to be useful in obtaining more DNA profiles from low-template samples, it is not a change to the PCR cycling conditions, but rather an extra repeat of the same uniform amplification conditions.

The most significant change to PCR cycling conditions for DNA profiling came with the introduction of Rapid DNA. The temperatures of the fundamental stages of the PCR are largely the same in Rapid PCR as they are in traditional STR PCR programs; however, it is the timing of the steps that changes substantially. The general Rapid DNA kit has a specialized chemistry and a PCR protocol involving enzyme activation (initialization) at 96 °C for 60 s, followed by 28 cycles of denaturation at 96 °C for 5 s and a combined annealing and extension step at 60 °C for 40 s, before a final extension step (hold) at 60 °C for 8 min [73,74,79,93,94]. These timing differences are further accentuated when the cycling conditions of traditional kits and Rapid DNA kits are directly compared, as shown in Table 1. These Rapid PCR programs are valuable to forensic investigations as they allow informative DNA profiles to be generated quickly, in the field at remote locations, and by personnel with little training. However, it is important to highlight that Rapid DNA has a specific range of applications, and currently has only had varying success with the sub-optimal samples that are typically encountered in casework [95]. Thus, a major limitation of Rapid DNA cycling conditions is that they generally require high-quality samples (i.e., reference samples) to generate good-quality, informative DNA profiles [96].

While Rapid DNA has successfully proven that changes can be made to elements of a PCR program, it is important to highlight that the changes used remain non-variant across the entire program. While this non-variance is a common feature of PCR programs within forensic genetics, there have been variations in cycling conditions through the generations of traditional STR PCR kits, as can be seen in Table 1. This is perhaps most notable when looking at the recommended cycling conditions for PowerPlex^®^ 2.1 and PowerPlex^®^ 16 systems (supplied by the Promega Corporation, Madison, WI, USA) and the VeriFiler^TM^ Plus system (available from ThermoFisher Scientific), which have a decreasing denaturation temperature and annealing/extension temperature after the first few cycles, respectively (Table 1).

**Table 1:** PCR cycling conditions recommended by commercially available traditional and rapid STR kits and the total reaction volumes.

STR Kit	Type	Year	Cycling Conditions			Total Cycles	Reaction Volume
			Denaturation	Annealing	Extension		
PowerPlex1.1 [97] and PowerPlex 2.1 [98]	Traditional	1997	94 °C 30 s (10 cycles) 90 °C 30 s (20 cycles)	60 °C 30 s	70 °C 45 s	30	22.5 μL
SGM Plus [99]	Traditional	1999	94 °C 1 min	59 °C 1 min	72 °C 1 min	28	50 μL
PowerPlex 16 [100]	Traditional	2001	94 °C 30 s (10 cycles) 90 °C 30 s (22 cycles)	60 °C 30 s	70 °C 45 s	32	25 μL
AmpFlSTR Identifiler [101]	Traditional	2001	94 °C 1 min	59 °C 1 min	72 °C 1 min	28	26 μL
MiniFiler [102]	Traditional	2007	94 °C 20 s	59 °C 2 min	72 °C 1 min	30	25 μL
AmpFlSTR Identifiler Plus [103]	Traditional	2010	94 °C 20 s	59 °C 3 min		28–29	25 μL
AmpFlSTR NGM Select Express [104]	Rapid	2011	94 °C 3 s	59 C 16 s	65 °C 29 s	25–28	25 μL
PowerPlex 21 [32]	Traditional	2012	94 °C 10 s	59 °C 1 min	72 °C 30 s	30	25 μL
GlobalFiler and GlobalFiler IQC [35]	Traditional	2013	94 °C 10 s	59 °C 90 s		29–30	25 μL
GlobalFiler Express [105]	Rapid	2015	94 °C 3 s	60 °C 60 s		25–28	15 μL
VeriFiler Plus [106]	Traditional	2018	96 °C 10 s	62 °C 90 s (2 cycles) 59 °C 90 s (27 cycles)		29	25 μL
VeriFiler Express [107]	Rapid	2021	96 °C 10 s	59 C 16 s	65 °C 29 s	25–28	25 μL

The concept of unchanging PCR conditions makes little sense when the conditions within the tube at the start of the PCR are compared to the conditions at the end of the reaction. The variation in reaction conditions across a PCR run is based on the amount of initial DNA template compared to the number of amplicons present at the culmination of the PCR process and the processivity of the enzyme. At the first cycle of PCR, the amount of template may be small (if 10 cells, then 20 priming sites for each primer when amplifying autosomal STRs), yet the enzyme is at its most active. After 28 cycles of PCR, the number of amplicons will be in the billions, yet the enzyme will have lost much of its activity. The extension time in the initial cycles could be reduced given the activity of the enzyme and the small amount of template, but increased as the template increases and enzyme processivity decreases. Equally, the denaturation temperature could decrease if the amplicons are short in length and there are no longer sections of chromosomal DNA.

The evolution of PCR for DNA profiling has slowed in recent years, despite the characteristic issues of degraded and inhibited samples still being present. Many changes have been made to elements of DNA profiling beyond the PCR programs, and yet the issues of these challenging sample types still remain.

#### 3.1.2. Mitochondrial DNA Testing

Parallel to the advent of STR amplification for DNA profiling, mitochondrial DNA (mtDNA) amplification for forensic analysis was also established in the early 1990s. The targeted amplification of three relatively small hypervariable regions in the human mitochondrial genome, HV1, HV2 and HV3 [108,109], provides an alternative means of identification when traditional sources of nuclear DNA (i.e., body fluids and tissues) are no longer available [17]. The power of mtDNA to provide information where standard methods fail comes from the fact that there can be thousands of mitochondria in each cell, each with 2 to 10 copies of mtDNA. However, unlike the nuclear DNA targeted for STR profiling, mtDNA is only inherited maternally. Thus, mtDNA analysis has the ability to identify maternally related individuals (i.e., siblings), as well as distinguish between individuals from different maternal lines [110]. This means the uses for mtDNA investigations are specialized, rather than being the primary tool for identification. The value of these markers is most evident where mtDNA amplification and analysis has been crucial to the identification of missing persons [18], unidentified human remains [111,112,113,114] and disaster victims [115].

As a result of mtDNA techniques developing alongside STR PCR methods, the PCR process used to amplify the mtDNA targets for forensic analysis is very similar. However, the reasoning behind the conservation of PCR cycling conditions for STR profiling and mtDNA analysis are not the same. While one of the main factors influencing PCR cycling conditions for STR profiling is the number of targets needed to generate highly discriminatory profiles, the cycling conditions used for mtDNA amplification do not face these same restrictions, as far fewer regions are targeted. This is because, prior to the use of massively parallel sequencing (MPS) platforms, all mtDNA amplifications were performed with one pair of primers, as it was not possible to multiplex if the amplicons were going to progress to Sanger sequencing. The smaller number of primers required for mtDNA analysis means there is a flexibility in the annealing stage that is not afforded in STR or SNP amplification. Most challenges of mtDNA analysis lie beyond the PCR process. The inherent variability in the mitochondrial genome of an individual, known as heteroplasmy [116,117,118], and the increased susceptibility for contamination to occur during the mtDNA extraction process [119] provides substantial challenges for mtDNA interpretation. However, the conservation of cycling conditions that can be seen in mtDNA PCR protocols used over the last three decades (Table 2) can instead be attributed to the push for reliability and reproducibility in the technique. Unlike STR PCR, the conservation of mtDNA amplification processes is likely a result of forensic laboratories adhering to the guidelines put forward by the International Society for Forensic Genetics for mtDNA typing [119,120] as a means of quality control. The need for quality control in mtDNA analysis is intensified due to of the lack of commercially available PCR kits. All commercially available STR PCR kits come with recommended cycling conditions that have been extensively validated by the manufacturer. This means laboratories only need to verify the kit prior to implementation in casework. The lack of commercial mtDNA kits means there is not the same pre-validation behind the PCR conditions used, allowing much more variability in the cycling conditions that can be used for mtDNA amplification between laboratories.

Due to the low-template and degraded nature of samples commonly submitted for mtDNA analysis (i.e., bone, teeth and hair), some changes have been made to the number of PCR cycles used to amplify mtDNA targets. The number of additional cycles ranges from 2–12 cycles higher than STR PCR in an effort to maximize the amount of mitochondrial data obtained from these challenging samples (Table 2). However, as mentioned earlier, the addition of extra PCR cycles is not actually a change to the cycling conditions themselves, but rather a repeat of the same uniform conditions. The shorter denaturation, annealing and extension steps used in current mtDNA PCR programs may be attributed to improved PCR instruments and reagents; however, it also provides some evidence to support the suggestion that the timing of the stages can be shortened without affecting target amplification. The challenges that heteroplasmy and contamination continue to present for mtDNA analysis are not dependent on PCR dynamics, and so cannot be remedied by altering the PCR process. Nevertheless, the potential to optimize mtDNA amplification methods by using non-uniform PCR programs that change as the conditions within the PCR tube change still exists.

#### 3.1.3. Single Nucleotide Polymorphism Analysis

While the amplification of single nucleotide polymorphisms (SNPs) for forensic analysis was used in casework before STR profiling [15], the issues associated with creating SNP multiplexes and the substantially lower discrimination powers afforded by SNP profiles saw STR profiling become the primary technique for human identification [126]. However, SNP analysis continued to be developed in response to the need for faster and more reliable techniques for the amplification of highly degraded and trace samples. In addition, there are abundant SNPs on both nuclear and mtDNA, allowing profiling using this technology to target both DNA types [127,128].

In recent years, SNP analysis has emerged as a major player in the world of forensic genetics due to the broad range of information it can provide from small amplicons [129,130,131]. This is because the single nucleotide variations that are targeted by SNP analysis are highly abundant in the human genome as a result of mutagenesis [129]. As the number of SNPs identified to be highly variable in the human genome continues to increase, so too does the power of discrimination between genomes when it is applied to forensic investigation. Given the abundance of forensically relevant SNPs, they are divided into five main categories based on the type of information they can provide to investigators. These categories are identity-informative SNPs (iiSNPS), lineage-informative SNPs (liSNPs), ancestry-informative SNPs (aiSNPs), phenotype-informative SNPs (pISNPs) and pharmacogenetic SNPs [31,132,133]. The combination of these SNPs can give insight into the biogeographical ancestry (BGA) and externally visible characteristics (EVCs) of an individual, in addition to the human identification capabilities that are afforded by STR and mtDNA assays [134], which highlights the value of SNP analysis to forensic investigations.

There are clear similarities between the previously mentioned mtDNA and STR PCR programs and the cycling conditions used for SNP analysis (Table 3). However, this is not surprising, given that SNP multiplex was developed in an attempt to overcome some of the challenges associated with the analysis of degraded and low-template mtDNA and STR samples. The variation in PCR cycling conditions between SNP assays (Table 3) can mostly be attributed to the different combinations of SNP targets that are used, and thus the different combinations of primers that require different annealing conditions to ensure all targets are amplified equally and efficiently. While early assays targeted 12 individual SNPs [135], more recently developed SNP panels, such as those used for modern forensic investigative genetic genealogy (FIGG) applications, target up to 1 million individual SNPs in a single assay [136]. The addition of large numbers of new SNP targets is also the reason why the cycling conditions have been highly conserved, as all targets need to be amplified in a balanced manner, and thus, have highly stringent annealing conditions. The increased number of PCR cycles can once again be attributed to the push for increased amounts of genetic data to be obtained from SNP analysis, with many samples submitted for SNP analysis containing low amounts of DNA or highly degraded material [127,128]. However, as previously noted, the variation in cycle number only signifies extra repeats of the same uniform PCR conditions and not actual changes to the cycling conditions themselves. As with STR profiling, changes have been made to other elements of the PCR process (i.e., commercial buffers, enzymes, instruments) to help further improve the amplification efficiency, and thus the overall quality, of the SNP data obtained [137].

Despite the success of SNP analysis in recent years, the technique has begun to move away from traditional target amplification methods, such as PCR, to new technologies that sequence the DNA molecules directly without any need for PCR. Such technologies, which include the Oxford Nanopore minION [142], Illumina NovaSeq 6000 [143] and silicon microchips [67], provide higher-throughput methods for SNP analysis using MPS platforms. Therefore, while the potential does exist for SNP PCR programs to be further optimized by employing gradually changing cycling conditions that account for the changes in enzyme activity across a run, the shift in focus from traditional Sanger sequencing platforms that require PCR to next generation technologies that do not indicates that perhaps the continued evolution of SNP analysis lies beyond the PCR process.

While the PCR processes used for STR profiling, mtDNA testing and SNP analysis have all evolved substantially since they were first introduced to forensic science, the once-rapid evolution has slowed substantially in recent years. Though this is not necessarily an issue for mtDNA and SNP analysis, the characteristic issues of interpreting degraded and inhibited samples for STR profiling still present significant limitations for DNA profiling. However, other disciplines, such as medical science, have continued to see rapid evolution of the PCR process, and as a result, highly successful PCR variants have been developed and integrated into their workflows.

### 3.2. Evolution of PCR Cycling Conditions in Other Disciplines

Touchdown PCR differs from the traditional PCR method as it involves a stepwise decrease in annealing temperature in each cycle of PCR [144]. This process developed in response to a demand for increased primer binding specificity in clinical research [144,145,146]. The annealing temperature in the first PCR cycle is substantially higher than the temperature at which the primers will melt, which is approximately 60–66 °C [145,146]. This reduces the amount of off-target primer binding due to their stringent binding requirements only allowing them to bind to exactly complementary regions on the target DNA at such high temperatures [145,146]. With each cycle, the temperature of denaturation decreases (typically by a standard amount such as 1 °C) as the highly specific regions amplified in early cycles become template DNA strands of only the target regions [145,146]. Importantly, touchdown PCR has been developed for both multiplex and uniplex reactions, but it has yet to be integrated into forensic casework, due in part to laboratories following the PCR cycling conditions provided by the manufacturers of the DNA profiling kits for standard PCR setups [145,146], and the fact that primers within these profiling kits are all designed to work optimally within a small range of temperature.

Similarly, gradient PCR was developed to aid in the determination of the optimal annealing temperature to increase primer specificity during PCR [147]. However, it is not technically a modification of the traditional PCR program; rather, it relies on changing the annealing temperatures by using a heating block that possesses a temperature gradient across its surface during the annealing stages. The determination of the optimal annealing temperature has played an important role in clinical pathology, where it has aided in the development of PCR protocols for SARS-CoV-2 (COVID-19) [148]. Additionally, the technique can also be applied to optimize the denaturation and extension phases of PCR, but it has only been tested for a single amplification, rather than a multiplex [84,149]. Thus, research into the application of gradient PCR to multiplex systems must be conducted to determine the viability of integrating this technique into forensic practice.

## 4. Recent Developments in PCR for DNA Profiling

### 4.1. Increased Speed

The significant increases in reaction speed and sample throughput that have been seen over the last three decades can largely be attributed to the improved technology within PCR instruments. In the early days of PCR, the process was highly labor-intensive, requiring the manual movement of individual tubes between water baths and the addition of DNA polymerase at the beginning of each cycle (as the enzymes were not yet thermostable) [2]. The production of “Mr. Cycle” in 1987 [150], the first automated thermal cycler that heated and cooled using a metal block, and the use of a thermostable DNA polymerase (*Thermus aquaticus*) in 1988 [5], are the two features that pushed PCR into a new era. As significant advancements in technology were made, the PCR process became faster, with mineral oil being replaced by heated lids (preventing sample evaporation and condensation) and bulky plumbing compressors traded for Peltier blocks with temperature control algorithms that can heat and cool rapidly [150,151]. The significant increase in machine ramp rates and heat dispersal rates within the PCR process in recent years has allowed the speed and throughput of PCR to increase substantially. 

Technological advancements in the thermal cyclers resulted in improved PCR instrument ramp rates and faster sample heating and cooling, which have helped speed up the time required to generate DNA profiles considerably. In conjunction, the identification of mutant DNA polymerases with improved processivity (amplification efficiency) and their application to forensic analysis has helped to speed up PCR [60,152,153,154]. Zhang et al. [154] developed a PCR enhancer cocktail containing non-ionic detergent, l-carnitine and d-(+)-trehalose, which worked to improve the performances of both commercially available Taq polymerases (i.e., AmpliTaq Gold and HotStarTaq Plus) and mutant DNA polymerases. While there has been substantial evidence that mutant polymerases can overcome inhibition more effectively than AmpliTaq Gold and amplify DNA more efficiently [60,152,153,154], they are yet to be adopted into commercially available STR kits. However, it is important to note that while DNA polymerases with increased processivity have successfully been used for DNA profiling, the challenges associated with sub-optimal samples still have not been overcome using these methods.

The largest alteration to PCR cycling conditions in forensic science came with the introduction of Rapid DNA instruments to laboratory workflows. While the features and importance of Rapid DNA have already been discussed, it is important to emphasize that Rapid DNA was designed for high-quality samples (i.e., reference samples) and has only had varying success when used with sub-optimal samples [95]. This highlights an important limitation of Rapid DNA: it requires good-quality DNA samples to generate informative DNA profiles [96].

### 4.2. Increased Sensitivity and Discrimination Power

The five-fold decrease in the amount of starting DNA required to generate probative STR profiles [19,32,35,99,103,106] in the last 30 years can be attributed to a number of factors: improved commercial DNA extraction processes ensuring inhibitors are removed, better buffer components in the STR PCR kits, and the increased number of STRs targeted for DNA profiling. The early multiplex PCRs for DNA profiling targeted only three loci, but quickly increased to seven loci, resulting in match probabilities of 1 in 50 million [54]. The identification and designation of the 13 core CODIS loci (USA) and 12 European Standard Set (ESS) loci then increased the possible match probability for informative DNA profiles to exceed 1 in 1 trillion [55,155]. The substantial increase in discrimination power of the widely used STR kits can be directly attributed to the increased number of loci targeted in recent years (see Table 4). The introduction of new, highly polymorphic loci, such as SE33, which has a high mutation rate of 0.64% [156,157,158], to the core CODIS and ESS loci in STR kits, as well as the increase in discrimination power that they afforded, have been key driving factors of DNA profiling in the last three decades. However, this push for increased sensitivity and discrimination powers has diminished in recent years as match probabilities now considerably exceed the total global population by many orders of magnitude. While the amount of DNA required to generate informative DNA profiles has decreased and the discrimination powers from good-quality genetic material has increased, the match probabilities (and likelihood ratios) of sub-optimal STR profiles generated from low-template and degraded samples are still limited by poor success rates.

### 4.3. Optimization of Commercially Available Kits

With the introduction of the new STR kit AmpFlSTR Minifiler^TM^ by Applied Biosystems (Foster City, USA) in 2007 came a new and improved PCR buffer [23,102]. This new buffer allowed the pH of the amplification reaction to be maintained through the presence of potassium (K^+^) and ammonium (NH_4_^+^) ions, which work to optimize and sustain the processivity of the DNA polymerase [159,160]. The identification of inhibitor-tolerant DNA polymerases [60,152,153] and their application to STR PCR saw a substantial increase in the number of complete DNA profiles generated from samples that contain PCR inhibitors [66]. Through the use of improved buffers, DNA polymerases and primers in commercially available STR kits, the amount of PCR product generated from samples has increased, and thus, the chances of producing informative DNA profiles from trace, inhibited or degraded samples have also increased.

While the success of generating DNA profiles from challenging samples has increased substantially in recent years, the quality of the profiles generated can still be less than ideal. The characteristic features of sub-optimal samples, such as small peak heights, allelic drop-in and drop-out, inhibited locus amplification, and heterozygote imbalance, are still present in the DNA profiles generated from these samples [23,36,57,161,162]. Therefore, while the optimization of commercial STR kit components has improved the ability to generate DNA profiles from trace materials, the presence of stochastic effects and the challenges they pose during profile interpretation still have not been overcome [23,36,52,57,161].

### 4.4. PCR Amplification Kinetics

The advent of fully quantitative PCR (qPCR) provided insight into how the environment within a PCR tube changes across the course of the reaction [51]. This insight has allowed researchers to identify how different components of the reaction, such as the primer concentration, magnesium (Mg^2+^) concentration, pH, DNA polymerase processivity and polymerase inhibitor tolerance, affect the kinetic behavior of the amplification [64,68,163]. Furthermore, the amount of template DNA present, and the quality of this DNA, have been found to affect PCR kinetics [68]. In addition to furthering our understanding of the amplification process, qPCR has allowed a range of PCR inhibitors to be identified, such as hemoglobin [65,164,165,166], proteases [167], calcium [65,167] and ethylenediaminetetraacetic acid (EDTA) [168], along with the mechanisms through which they inhibit DNA amplification [169].

The significant developments in our understanding of PCR kinetics and how they change as a reaction progresses have provided critical insight into the mechanisms of DNA amplification. Importantly, this level of understanding was not something that was known when PCR was developed as a tool for DNA profiling, and while the process has been effectively validated over the last few decades [170], there are likely ways that it can be made more efficient. Due to dramatic improvements in qPCR system sensitivity and our deeper understanding of how the composition within a PCR tube changes across the course of a PCR program, the opportunity now exists to monitor how amplification kinetics change as specific elements of the PCR process are altered. The effects of changes to PCR cycling conditions, such as small changes to timing and temperature, can be carefully monitored, and their influence on amplification efficiency can be clearly defined. Furthermore, the cycling conditions that are found to increase amplification efficiency could then be used to improve the amplification of degraded and/or inhibited samples. The concept here would be to revisit the PCR cycling conditions and adjust the first cycles to account for low template DNA, and then alter the cycling conditions in stages through to the last cycle where enzyme processivity has slowed dramatically.

## 5. Recent Developments in PCR beyond Forensics

Lab-on-a-Chip devices for biological analyses outside laboratories and their integration with intelligent computer systems in recent years have significantly improved the ability to manipulate and monitor the PCR process [171]. The development of electrowetting-on-dielectric (EWOD) digital microfluidics devices came as an alternative to the time-, labor- and cost-intensive procedures required to study biomolecular interactions for clinical diagnostics and pathogen detection [171,172]. EWOD devices utilize fluorescent feedback detected through optical sensors to monitor reactions in real-time. Since they were first pioneered, EWOD systems have continued to evolve, with detectors becoming more sensitive, and only picolitres of sample are required for successful quantitative analysis [173,174]. The rapid heating and cooling rates, low reagent consumption, high sensitivity, high throughput, portability, and short run times of the EWOD platforms makes them ideal for PCR assays [175,176,177]. Additionally, the multidisciplinary collaboration in recent years between biochemists, engineers and computer scientists has enabled the development of high-level biological programming languages that can be integrated into microfluidic devices to detect and monitor reactions in real-time, and control the devices using feedback loops [177,178]. Importantly, multiplex PCR procedures have been designed and optimized for these microfluidic systems, and they have been proven to have high amplification efficiencies [175,179,180]. These high amplification efficiencies can be attributed to the intelligent computer software developed to make decisions in real time based on the fluorescence feedback obtained [177,178]. Multiple different DNA samples have also successfully been amplified concurrently and monitored in real-time using a digital microfluidic platform [179]. The development of microfluidic multiplex PCR assays highlights the viability and value of integrating intelligent computer systems into traditional biochemical processes to optimize them based on real-time feedback. The potential exists for such a system to be developed for forensic DNA profiling that could monitor amplification efficiency in real time and alter the PCR cycling conditions to generate an ideal amount of amplified product for DNA profiling.

## 6. Remaining Challenges in PCR for DNA Profiling

While the PCR programs used for STR PCR have been shown over the years to be reliable and robust, their largest pitfall is that they require large quantities of good-quality genetic material to produce informative DNA profiles. The development and improvement of commercially available PCR kits and instruments in recent years has significantly improved the speed and sensitivity of the technique, but the once-strong drive for improvement has slowed despite the characteristic issues of degraded and inhibited samples still being present. Many changes have been made to elements of DNA profiling beyond the PCR programs; however, they have not been overly successful in improving our ability to profile challenging DNA samples. With trace DNA samples being one of the most commonly submitted sample types in many jurisdictions, any improvement in the success rate of DNA profiling these samples would be valuable to forensic investigations. Rapid DNA and commercial STR kits have provided evidence to suggest that changes to the timing and temperatures used for PCR (either in a univariant or gradient form) could be made to increase amplification efficiency and improve the quality of DNA profiles produced. This begs the question: what changes can be made to PCR cycling conditions to finally improve DNA profiling success rates from challenging samples?

Gradual changes have been successfully made to the denaturation and polymerization steps of a PCR without compromising the evidentiary value of the DNA profiles generated in a recent proof-of-concept study [181]. While this was done using ideal amounts of pristine DNA, it does suggest that the PCR process could be altered to improve the DNA profiles obtained from characteristically challenging samples. Given the success of incorporating machine learning into biological processes in microfluidics [177,178], an exciting future direction would be to integrate such a system into the process of setting PCR cycling conditions for DNA profiling. Such a system would require a means to monitor the PCR as it progresses through real-time fluorescence feedback. A method to obtain this real-time fluorescence feedback was recently suggested that involved combining a standard STR reaction and a qPCR into a single tube [182]. Furthermore, the smart system would also require a means to use the fluorescence information in a machine learning algorithm to adjust the cycling conditions on a per-cycle basis, and a means to do this on a per-sample basis. Such work is in its inception, but may provide exciting avenues in the future.

## Figures and Tables

**Table 2:** PCR cycling conditions used to amplify mitochondrial DNA and the total reaction volumes. “N/A” is used where the temperature of the annealing step is varied depending on the primers used in each reaction.

Protocol	Year	Cycling Conditions			Total Cycles	Reaction Volume
		Denaturation	Annealing	Extension		
Stoneking et al. [16]	1991	94 °C 45 s	56 °C 1 min	74 °C 1 min	30	100 μL
Sullivan et al. [121]	1992	94 °C 45 s	50 °C 1 min	72 °C 3 min	32	25 μL
Handt et al. [122]	1996	92 °C 50 s	N/A 50 s	72 °C 50 s	40	40 μL
Berger and Parson [123]	2009	95 °C 15 s	57 °C 10 s	72 °C 20 s	39	40 μL
Kim et al. [124]	2013	95 °C 20 s	55 °C 60 s	72 °C 30 s	42	25 μL
Cooley [125]	2023	94 °C 20 s	56 °C 20 s	72 °C 30 s	38	40 μL

**Table 3:** PCR cycling conditions used to amplify single nucleotide polymorphisms (SNPs) and the total reaction volumes.

Protocol	Year	SNP Type	Cycling Conditions			Total Cycles	Reaction Volume
			Denaturation	Annealing	Extension		
Tully et al. [135]	1996	Mitochondrial	94 °C 30 s	57 °C 30 s	72 °C 90 s	35	50 μL
Ahmadian et al. [138]	2000	Nuclear	94 °C 1 min	50 °C 40 s	72 °C 1 min	35	50 μL
Andréasson et al. [127]	2001	Mitochondrial	95 °C 30 s	54 °C or 60 °C 45 s	72 °C 1 min	40	100 μL
Inagaki et al. [139]	2004	Nuclear	96 °C 10 s	50 °C 5 s	60 °C 30 s	25	5 μL
Divne and Allen [140]	2005	Nuclear and Mitochondrial	94 °C 30 s	55 °C 30 s	72 °C 30 s	35	50 μL
McNevin et al. [141]	2011	Mitochondrial	94 °C 1 min	56 °C 1 min	72 °C 1 min	30	25 μL

**Table 4:** Number of loci targeted by commercially available STR Kits.

STR Kit	Year	Loci Targeted	Total
PowerPlex1.1 [97]	1997	Amelogenin, CSF1PO, D5S818, D7S820, D13S317, D16S539, TH01, TPOX, vWA	9
PowerPlex 2.1 [98]	1997	D3S1358, D8S1179, D18S51, D21S11, FGA, Penta E, TH01, TPOX, vWA	9
SGM Plus [99]	1999	Amelogenin, D2S1338, D3S1358, D8S1179, D16S539, D18S51, D21S11, D19S433, FGA, TH01, vWA	11
PowerPlex 16 [100]	2001	Amelogenin, CSF1PO, D3S1358, D5S818, D7S820, D8S1179, D13S317, D16S539, D18S51, D21S11, FGA, Penta E, Penta D, TH01, TPOX, vWA	16
MiniFiler [102]	2007	Amelogenin, CSF1PO, D7S820, D13S317, D16S539, D18S51, D21S11, D2S1338, FGA	9
AmpFlSTR Identifiler Plus [103]	2010	Amelogenin, CSF1PO, D2S1338, D3S1358, D5S818, D7S820, D8S1179, D13S317, D16S539, D18S51, D19S433, D21S11, FGA, TH01, TPOX, vWA	16
PowerPlex 21 [32]	2012	Amelogenin, CSF1PO, D1S1656, D2S1338, D3S1358, D5S818, D6S1043, D7S820, D8S1179, D12S391, D13S317, D16S539, D18S51, D19S433, D21S11, FGA, Penta D, Penta E, TH01, TPOX, vWA	21
GlobalFiler and GlobalFiler IQC [35]	2013	Amelogenin, CSF1PO, D1S1656, D2S441, D2S1338, D3S1358, D5S818, D7S820, D8S1179, D10S1248, D12S391, D13S317, D16S539, D18S51, D19S433, D21S11, D22S1045, DYS391, FGA, TH01, TPOX, SE33, vWA, Yindel	24
VeriFiler Plus [106]	2018	Amelogenin, CSF1PO, D1S1656, D2S441, D2S1338, D3S1358, D5S818, D6S1043, D7S820, D8S1179, D10S1248, D12S391, D13S317, D16S539, D18S51, D19S433, D21S11, D22S1045, FGA, Penta E, Penta D, TH01, TPOX, vWA, Yindel	25

## Data Availability

Not applicable.
